# Framing the Raccoon: Discourse, Governance, and Ethics in Urban Wildlife Management in Germany

**DOI:** 10.3390/ani16192996

**Published:** 2026-09-23

**Authors:** Birkan Taş

**Affiliations:** UPF-Centre for Animal Ethics (UPF-CAE), Department of Communication, Universitat Pompeu Fabra, 08018 Barcelona, Spain; birkanka@gmail.com

**Keywords:** raccoons, invasive species, urban wildlife, frame analysis, critical discourse analysis, animal welfare, wildlife management, Germany

## Abstract

Raccoons (*Procyon lotor*) are classified as an invasive alien species of European Union concern, yet their governance involves ecological, legal, and ethical considerations. This study examines how raccoons are framed and governed in Germany, with particular attention to Berlin and Kassel/Hesse, and how these framings legitimise different interventions. Frame analysis and critical discourse analysis were applied to 39 governmental, parliamentary, public-information, and organisational documents. Four recurring and overlapping frames were identified: invasive threat, urban conflict/pest, manageable population, and cohabiting neighbour. These frames define harm and allocate responsibility differently, legitimising responses ranging from prevention and coexistence to hunting and fertility control. They also differ in which forms of animal suffering they make visible or obscure. Even non-lethal fertility control could reproduce a control-oriented logic when the need to reduce raccoon populations was assumed rather than justified. Raccoon governance is therefore shaped not only by ecological evidence and legal obligations but also by judgments concerning risk, order, belonging, care, and control. The analysis shows how invasive classification structures evidential and moral priorities, foregrounding population-level ecological harms while often subordinating human responsibility and the interests of individual raccoons. It argues that invasive status alone cannot establish either the necessity or the ethical acceptability of intervention: documented local harms, expected benefits, harms imposed on individual animals, feasible alternatives, and human responsibility must all be made explicit, while non-intervention must remain possible where serious harm has not been demonstrated.

## Simple Summary

Within dominant conservation policy, “invasive alien species” are organisms introduced by humans outside their historical range and judged capable of causing ecological or other harm. Raccoons (*Procyon lotor*) entered Germany through human-mediated introductions and subsequently also spread through natural dispersal. They are classified as an invasive alien species of Union concern. Member states are required to address widely spread populations of listed species. In Germany, raccoons are common in many cities, but their presence is understood in different ways, and there is disagreement over whether—and under what conditions—intervention is justified. This study examined 39 public documents, with particular attention to Berlin and Kassel/Hesse. Raccoons were described as threats to native wildlife, sources of urban conflict, populations to be controlled, or urban neighbours. These views supported different responses, including prevention, hunting, sterilisation, and coexistence. The findings show that decisions about whether and how to intervene are shaped not only by scientific evidence and law, but also by different ideas about harm, responsibility, animal welfare, and coexistence. The invasive label is therefore not ethically neutral: it can make threats to species and populations more visible while placing less attention on human responsibility and the experiences of individual raccoons.

## 1. Introduction

Within the dominant conservation-policy framework, invasive alien species are identified as one of the major direct drivers of biodiversity loss, alongside land- and sea-use change, direct exploitation, climate change, and pollution [1]. This framework associates human-mediated movement beyond historically defined ranges with ecological harm, economic costs, infrastructure damage, and risks to human and animal health [2]. It is institutionalised in Regulation (EU) No. 1143/2014, which requires prevention, rapid eradication following early detection, and management of listed species already widely spread within a Member State [3]. This article treats these classifications and obligations as objects of analysis rather than as neutral descriptions that determine in advance how particular animals should be understood or treated.

These obligations neither make all introduced populations ecologically equivalent nor prescribe a single response. Impact assessments depend on ecological endpoints, spatial and temporal scale, available evidence, and the treatment of uncertainty [4,5]. In particular, management decisions may have to address harms that are incompletely demonstrated, spatially variable, or projected on the basis of anticipated ecological risk. Decisions about whether and how to intervene also involve judgments about effectiveness, feasibility, proportionality, and the distribution of harms among biodiversity, human communities, and individual animals. For widely spread species, EU law permits lethal and non-lethal measures but requires them to be appropriate to local circumstances and to spare animals avoidable pain, distress, or suffering [3]. Listing therefore creates an obligation to act without determining a single form of intervention.

The dominant framework and its terminology are themselves contested. Some studies have argued that introduced species are disproportionately framed in negative terms, while others maintain that such framing reflects documented ecological risks and the difficulty of predicting which introductions will become harmful [6,7]. The category “invasive” nevertheless performs both descriptive and normative work: it identifies origin and possible ecological effects while also helping to define which animals belong, which harms receive priority, and which interventions appear legitimate. Animal ethics therefore introduces more than an additional welfare criterion for selecting management methods. It asks whether non-native origin can reduce the perceived moral status of individual animals and make their capture, reproductive control, or killing appear acceptable before the circumstances and harms of a particular intervention have been examined [8].

The northern raccoon (*Procyon lotor*) provides a particularly revealing case through which to examine these tensions. Native to North America, raccoons became established in Germany during the twentieth century through deliberate releases and escapes from captivity including fur-farming infrastructures [9,10]. Although their subsequent expansion also involved natural dispersal, their presence originated in human decisions and practices rather than in an autonomous animal “invasion.” Framing raccoons themselves as the source of the problem can therefore obscure the historical responsibility of the humans and institutions that introduced them. In 2016, raccoons were listed as an invasive alien species of Union concern [11]. They are now established across Germany and widespread in parts of Central and Western Europe [12].

Raccoons are omnivorous mesopredators whose behavioural flexibility, climbing ability, and use of human-provided food and shelter enable them to inhabit rural, peri-urban, and urban environments [12,13]. In cities, raccoon presence can generate concerns about noise, property damage, entry into buildings, possible zoonotic disease transmission, and impacts on native biodiversity. These concerns arise through interactions among raccoon behaviour, human practices, urban infrastructure, and local ecological conditions [14,15]. Raccoon governance therefore brings ecological, property, public-health, legal, and animal-welfare concerns into the same policy field.

Research has examined raccoon expansion, population genetics, ecological impacts, zoonotic risks, and management options [9,10,12,13,16,17,18]. Raccoon rabies has been documented only sporadically in Germany, with the cases reported in the cited literature occurring between 1990 and 2000 [16]. The raccoon roundworm *Baylisascaris procyonis* occurs regionally and can infect humans and other animals, although human disease is rare [16,17,18]. Canine distemper can affect raccoons and other susceptible carnivores but is not zoonotic [16]. Recent German research has also shown that perceptions of raccoon impacts and preferred management measures differ among regions and stakeholder groups [15,19]. Comparatively little attention, however, has been paid to how governmental, parliamentary, and organisational documents themselves construct raccoon-related problems: how they define harm, allocate responsibility, invoke scientific and legal authority, and make particular interventions appear legitimate.

This study addresses this gap through comparative qualitative document analysis combining frame analysis and critical discourse analysis of public documents concerning raccoon management in Germany, with particular attention to Berlin and Kassel/Hesse. It asks how raccoon-related issues are framed and how these framings justify or contest intervention through constructions of harm, responsibility, evidence, belonging, and animal welfare. The analysis identifies four overlapping frames—invasive threat, urban conflict/pest, manageable population, and cohabiting neighbour—that cut across actor groups and institutions and shape whether prevention, hunting, fertility control, or coexistence appear legitimate. Rather than asking how the invasive-species paradigm might be implemented more effectively, the study examines how public documents reproduce, modify, or contest its assumptions and how alternative animal-welfare and coexistence perspectives redistribute responsibility and moral attention. The analysis thereby shows how ecological evidence, legal classification, human responsibility, and the moral status of individual animals are brought together—or separated—in justifications for intervention.

## 2. Materials and Methods

### 2.1. Research Design

This study used comparative qualitative document analysis to examine how raccoons (*Procyon lotor*) and their management are constructed in German urban wildlife governance [20]. The comparison operated across Berlin and Kassel/Hesse, as well as across actor groups, document types, and time. Frame analysis was used to identify how documents defined problems, attributed causes and responsibility, made moral evaluations, and recommended treatments [21]. Following Entman, framing makes selected aspects of an issue more salient and is defined partly by what it includes and omits [21] (p. 54). Critical discourse analysis examined how language and institutional authority made particular interpretations and interventions appear credible, necessary, proportionate, or appropriate [22,23,24].

### 2.2. Case Selection and Corpus

Berlin and Kassel/Hesse were selected as two historically significant regions of raccoon establishment in Germany. Kassel is located near Lake Edersee, where raccoons were deliberately released in 1934, while Berlin lies within the broader eastern distribution region associated with escapes of raccoons from a fur farm in Wolfshagen, Brandenburg, in 1945 [9]. Although genetic research indicates at least four independent introduction events, the Hesse and Brandenburg populations form the two largest and most widespread genetic clusters and correspond to the two best-documented founder regions [9]. Their long-established raccoon populations provide a meaningful basis for comparing frames and justificatory patterns rather than ecological conditions or impact levels. Berlin is both a city and a federal state, whereas raccoon governance in Kassel is distributed between municipal authorities and the state of Hesse. The inclusion of Hessian parliamentary, regulatory, and administrative documents was therefore necessary to analyse decisions directly affecting raccoon management in Kassel. Both cases have established urban raccoon populations and active management controversies, but they differ in the interventions that became politically salient. The focus on Berlin and Kassel/Hesse reflects the study’s concern with urban governance rather than the full geographical distribution of raccoons.

The analytical corpus comprised 39 publicly available documents: 18 Berlin governmental and public-information documents, 15 Kassel/Hesse municipal, parliamentary, party, and regulatory documents, and six organisational or position documents. The materials included parliamentary questions and government responses, committee records, laws and administrative documents, municipal webpages and press releases, project descriptions, and statements issued by political parties, conservation organizations, hunting associations, wildlife-support organizations, and animal-welfare bodies. National organisational or federal documents were included where they directly addressed management controversies relevant to the cases or articulated positions that entered those debates. The comparison therefore examined variation both within and between the two cases rather than treating either as representing a single governance approach. Six additional legal, policy, and scientific sources were used to establish regulatory and factual context but were not coded or included in the corpus total. Journalistic sources were used only to reconstruct chronology or locate primary sources.

The search window extended from 1 January 2020 to 25 August 2026. The final corpus contained 34 dated documents published between 2022 and 2026 and five undated webpages available during the sampling period; their original publication dates could not be verified. The undated webpages were treated as materials available at the time of retrieval and were not used to infer chronological change. Searches were conducted in parliamentary databases and on governmental, municipal, and organisational websites, supplemented by targeted web searches. Search terms included Waschbär and Waschbären (raccoon/s), *Procyon lotor*, invasiv (invasive), Management, Jagd (hunting), Bejagung (hunting/control through hunting), Bestandsregulierung (population control), Sterilisation (sterilisation), Kastration (castration), Tierschutz (animal welfare), Artenschutz (species conservation), and Amphibien (amphibians), used separately and in combination. References and links within retrieved documents were followed iteratively. Sampling was purposive and aimed to capture variation in institutional position, actor type, and proposed intervention rather than exhaustive coverage or frequency estimates.

Documents were included when they: (1) contained a substantive raccoon-related passage or directly structured a documented raccoon-management decision; (2) concerned Berlin, Kassel/Hesse, or a national institutional position directly bearing on these cases; (3) addressed management, conflict prevention, hunting, fertility control, animal welfare, biodiversity, public health, or another relevant construction of harm; and (4) were published within the study period or, for undated webpages, publicly available during the sampling period.

### 2.3. Coding and Analytical Procedure

Each document was manually coded in a document-specific coding sheet. The complete document served as the contextual unit, while passages containing a distinct problem definition, causal attribution, evaluation, evidentiary claim, or management recommendation served as coding units. For committee records or documents addressing several topics, only raccoon-related passages and the surrounding context necessary for interpretation were coded.

The coding sheets recorded relevant German passages and page numbers, descriptive and interpretive codes, and analytical notes. Coding combined deductive categories derived from frame analysis with inductively developed thematic codes. Following Entman [21], passages were examined for problem definition, causal interpretation, moral evaluation, and treatment recommendation. Recurring codes included ecological harm, property damage, health risk, population growth, legal obligation, animal welfare, feeding, waste and building management, habitat degradation, public education, exclusion, monitoring, trapping, lethal removal, fertility control, care, and coexistence. Passages could receive multiple codes.

Critical discourse analysis was operationalised by examining labels applied to raccoons; constructions of agency and responsibility; expressions of certainty, uncertainty, and necessity; appeals to scientific and legal authority; and the interests or forms of harm made visible. Technical and population-level language was also noted where consequences for individual animals remained implicit. Potential absences were treated analytically only when the omitted issue was directly relevant to the document’s stated problem and recurred across comparable texts.

Codes were refined iteratively through repeated reading and comparison across documents. Recurring combinations of problem definitions, causal accounts, evaluations, and recommended treatments were subsequently grouped into higher-order frames. Frame assignment was non-exclusive, and contradictory or deviant passages were retained rather than absorbed into a dominant interpretation.

### 2.4. Use of Generative Artificial Intelligence

ChatGPT (OpenAI, GPT-5.6 Sol; accessed during August–September 2026) was used to assist with search-term development, identify potential sources for independent verification, and generate English working translations of German materials. All documents were independently retrieved from their original institutional sources and screened by the author. AI-generated translations were used only as comprehension aids and checked against the German originals; quoted translations were additionally checked using DeepL (web version; https://www.deepl.com/translator; accessed on 22 September 2026) and bilingual dictionaries. All quotations and analytically significant ecological and legal terms reproduced in the article were subsequently checked against the German sources by a native German speaker. ChatGPT did not determine document eligibility, assign final codes, or function as an independent coder. All final coding, interpretation, comparison, and verification were conducted by the author.

### 2.5. Reflexivity and Limitations

The analysis was conducted by one researcher and does not claim statistical representativeness or intercoder reliability. Analytical consistency was supported through repeated review of coded documents, comparison across contrasting positions and document types, and an audit trail of coding decisions.

The author has intermediate German proficiency (CEFR B1). German originals were read alongside English working translations, and analytically significant German terms and formulations were retained throughout the coding process. Coding and interpretation were repeatedly checked against the original texts, while quotations used in the manuscript were additionally verified using DeepL and bilingual dictionaries.

The corpus represents publicly articulated institutional positions rather than private intentions, public opinion, policy implementation, or the ecological effects of management. Claims were therefore interpreted in relation to their genre, institutional context, and recurrence across the corpus. The researcher’s interest in the ethical consequences of wildlife classification may increase sensitivity to animal-welfare exclusions. Welfare- and coexistence-oriented claims were therefore subjected to the same evidential and discursive scrutiny as conservation- and hunting-oriented claims.

## 3. Results

The corpus of 39 documents yielded four recurring, overlapping frames. Table 1 summarises their problem definitions, causal and moral logic, and favoured interventions; the subsections below report how each frame appeared in the documents themselves, how frame use aligned with actor category, and how one contested claim—raccoon predation on amphibians in Berlin—developed across the study period. Frames are reported descriptively here; their governance implications are addressed in Section 4.

### 3.1. Invasive Threat Frame

The invasive threat frame defined raccoons primarily through their effects on native biodiversity. It was most explicit in conservation, hunting, regulatory, and political documents. Zoonotic and Wildlife Ecological Impacts of Invasive Carnivores (ZOWIAC), for example, states that amphibians, reptiles, ground- and cavity-nesting birds, nest-dependent birds, and cave-dwelling mammals are “increasingly under pressure” (“zunehmend unter Druck”) and claims that predation “can lead to substantial population declines” (“zu erheblichen Bestandsrückgängen führen […] kann”) [25].

Within this frame, causal claims centred on predation and population expansion. The proposed responses included trapping, removal, targeted hunting near sensitive sites, and broader hunting provisions. ZOWIAC presented intensified hunting as unavoidable where preventive measures were insufficient [25]. Hunting-sector and Hessian state documents likewise described trapping, year-round hunting, and wildlife monitoring as practical and evidence-based forms of predator management [26,27,28]. Berlin’s 2025 hunting ordinance linked raccoons to risks for protected amphibian, reptile, and bird populations and authorised targeted hunting as a protective measure [29].

The frame also appeared in Berlin political and regulatory discourse. In the AfD inquiry, raccoons are described as an “alien and invasive species” (“gebietsfremde und invasive Art”) and an “opportunistic predator” (“opportunistischer Räuber”) threatening “our native fauna” (“unsere heimische Tierwelt”), including ground-nesting songbirds and amphibians [30]. The Senate qualified this claim: it described raccoons as an “additional factor impairing amphibian populations” (“zusätzlicher Faktor der Beeinträchtigung der Amphibienpopulationen”) and stated that there was no monitoring of feeding damage to bird clutches (“kein Monitoring zur Bemessung von Fraßschäden”) [30].

The strength of causal claims varied over time. In 2022, the Berlin Senate stated that raccoons could “locally worsen the situation for amphibians” (“lokal die Situation für Amphibien negativ verstärken”) but were not responsible for their dramatic decline [31]. In July 2025, by contrast, Stefan Richter of Stiftung Naturschutz Berlin described raccoons as “one of the greatest causes of amphibian loss” (“eine der größten Ursachen für den Verlust an Amphibien”) in Berlin [32]. The August 2025 hunting ordinance subsequently stated that raccoons “endanger birds and amphibians” (“gefährden Vögel und Amphibien”) and protected amphibian, reptile, and bird populations (“europarechtlich geschützte Amphibien-, Reptilien- und Vogelbestände”), while authorising “targeted hunting” (“gezielte Bejagung”) [29].

Causal certainty therefore ranged from qualified descriptions of additional local pressure to broader claims of biodiversity loss. The development of these claims in Berlin is examined in Section 3.6.

### 3.2. Urban Conflict/Pest Frame

The urban conflict/pest frame defined raccoon-related problems primarily through encounters in human spaces: entry into houses, waste access, noise, contamination, property damage, and unwanted proximity. Explicit designation of raccoons as pests was uncommon. Pest-like language appeared most clearly in formulations such as “situation and/or plague” (“aktuelle Lage und/oder Plage?”) in a 2024 Berlin parliamentary inquiry [33]. The same inquiry also referred to complaints about “devastation” (“Beschwerden über Verwüstungen”), reinforcing a damage-oriented representation of raccoon presence. The Senate did not confirm a citywide “plague” and noted the absence of systematic complaint or damage statistics.

Municipal documents more often framed raccoons as troublesome urban occupants than as pests. Kassel described the city as a “raccoon land of milk and honey” (“Waschbären-Schlaraffenland”) and linked high densities to food and shelter provided by humans [34]. Its 2025 public advice portrayed raccoons as “unwanted tenants” (“unliebsame Mieter”) whose entry into buildings could cause “extensive damage” (“große Schäden”) and “high costs” (“hohe Kosten”), while recommending structural “raccoon defence” (“Waschbär-Abwehr”) rather than general removal [35].

Pest-like associations were most explicit in a 2024 Kassel parliamentary question. It placed the “increasing, area-wide raccoon population” (“zunehmenden flächenhaften Waschbär-Population”) alongside an “infestation by pest rodents” (“Befall mit Schadnagern”) and asked how the city intended to “counteract” these conditions (“entgegenzuwirken”) [36]. Raccoons were not themselves called pests; rather, the juxtaposition situated them within a shared administrative vocabulary of unwanted urban infestation and control.

The attribution of responsibility varied within the frame. Municipal guidance linked conflicts to feeding, accessible waste, and buildings that provide shelter, thereby locating part of the cause in human behaviour and urban infrastructure [34,35,37,38].

### 3.3. Manageable Population Frame

The manageable population frame represented raccoon abundance and reproduction as objects of expert intervention. Its vocabulary centred on population control, reduction, monitoring, and the selection of effective methods. In Berlin, parliamentary debate referred explicitly to “raccoon management” (“Waschbärmanagement”), “local population control” (“lokale Populationskontrolle”), and sterilisation and castration (“Sterilisation und Kastration”) [39]. The Berlin sterilisation study similarly aimed to test the “sustainable reduction of the population size” (“nachhaltige Reduzierung der Populationsgröße”) and described sterilisation/castration as an “ethical, animal-welfare-compliant and effective sustainable method of population control” (“ethisches, tierschutzkonformes und effektives nachhaltiges Verfahren zur Populationskontrolle”) [40]. Its population-level effectiveness remained to be tested, while the legal permissibility of returning sterilised raccoons to their capture sites was contested.

The Kassel pilot applied a comparable logic through capture, sterilisation, and release. Municipal and project documents described its objectives as “stabilisation” (“Stabilisierung”) and “population reduction” (“Bestandsabbau”) [38,41,42,43]. Fertility control therefore made reproduction itself the mechanism through which raccoon numbers were to be governed.

Lethal management was articulated through the same managerial register. The Kassel CDU called for “scientifically grounded raccoon management” (“wissenschaftlich fundiertes Waschbär-Management”), “realistic targets for population reduction” (“realistischen Zielwerten zur Populationsreduktion”), and population control (“Bestandskontrolle”), while retaining shooting as a “last resort” (“als letztes Mittel”, “ultima ratio”) [44]. The Hessian Hunting Association challenged sterilisation as a “significant intervention on a vertebrate” (“erheblicher Eingriff an einem Wirbeltier”) and described its assumptions as “scientifically clearly refuted” (“wissenschaftlich eindeutig widerlegt”) [26]. The German Hunting Association instead described hunting as an “effective management measure” (“effektive Managementmaßnahme”) and argued that it should be promoted politically [45].

Across these positions, population control remained the shared premise: disagreement concerned which method should regulate raccoon numbers, how effectiveness should be established, and how welfare costs should be assessed. Animal welfare therefore functioned primarily as a constraint on the means of control rather than as a challenge to the prior assumption that control was necessary.

### 3.4. Cohabiting Neighbour Frame

The cohabiting neighbour frame represented raccoons as established urban wildlife whose presence requires negotiation rather than elimination. Within this frame, coexistence remained conditional and interventionist rather than unrestricted. Human-provided food, accessible buildings, and shelter were foregrounded as causes of conflict, shifting part of the responsibility from raccoons to human behaviour and urban infrastructure. Berlin documents promoted a “relaxed coexistence” (“entspanntes Nebeneinander”) through reducing food sources as well as sleeping and reproductive sites (“Nahrungsquellen sowie Schlaf- und Vermehrungsstätten”) [37,46]. Even after hunting provisions were expanded, the Senate retained the objective of “the least friction possible between humans and animals” (“möglichst reibungsloses Miteinander von Mensch und Tier”) [47,48].

This frame was particularly explicit in animal-welfare and conservation discourse. Berlin’s State Animal Welfare Commissioner described raccoons as “established” (“etablierte Spezies”) and stated that residents must accept “sharing our city with these fascinating animals” (“uns damit arrangieren, unsere Stadt … mit diesen faszinierenden Tieren zu teilen”) [49,50]. The Nature And Biodiversity Conservation Union (NABU) of Berlin similarly described raccoons as “neighbours” (“Nachbarn”), “co-inhabitants” (“Mitbewohner”), and “part of our wildlife fauna” (“Bestandteil unserer Wildtierfauna”), while stating that “raccoons were brought to Germany by humans—the animals are not responsible for the consequences” (“Waschbären wurden von Menschen nach Deutschland gebracht—für die Folgen können die Tiere nichts”) [51].

Kassel municipal guidance likewise treated permanent raccoon presence as a practical condition. Expelling raccoons from the city was described as “illusory” (“illusorisch”), while conflict reduction (“Konflikte … möglichst gering … halten”) was pursued through food restriction and structural exclusion [34,35,52]. The Kassel Greens further stressed that raccoons “did not come to Hesse voluntarily” (“nicht freiwillig nach Hessen gekommen”) [53]. These formulations accepted raccoon presence while redistributing part of the responsibility for conflict to human actions and environments.

### 3.5. Variation Across Actors, Places, and Time

The frames cut across actor categories and regions rather than corresponding to stable camps. Hunting organisations generally combined the invasive-threat and manageable-population frames, whereas animal-welfare organisations more often connected population management with individual welfare and non-lethal methods [26,40,45,54]. Berlin and Kassel/Hesse each combined acceptance of permanent raccoon presence with population-level intervention. Kassel paired coexistence-oriented municipal guidance with experimental fertility control, while political and hunting actors advocated stronger population control [34,41,42,43,44]. Berlin similarly moved from resource restriction and coexistence-oriented advice toward expanded hunting provisions from 2025, while continuing to describe lethal intervention as a last resort [29,37,46,47,48]. Actor, place, and time therefore affected the relative salience of the frames without producing a simple opposition between coexistence and control.

### 3.6. Changing Claims About Amphibian Decline in Berlin

Claims about raccoon effects on amphibians became stronger across the Berlin documents, although uncertainty remained. In 2022, the Senate stated that raccoons could “locally worsen the situation for amphibians” (“lokal die Situation für Amphibien negativ verstärken”) but were not responsible for their dramatic decline; deterioration of aquatic and terrestrial habitats was identified as the principal cause [31]. In September 2024, habitat destruction and fragmentation, climate change, and fungal disease were again identified as major pressures, while the raccoon was described as a possible “additional factor” (“zusätzlicher Faktor”) [33].

In July 2025, Stefan Richter of Stiftung Naturschutz (Nature Conservation Foundation) described raccoons as “one of the greatest causes of amphibian loss” (“eine der größten Ursachen für den Verlust an Amphibien”) in Berlin [32]. The August 2025 hunting ordinance subsequently stated that raccoons “endanger birds and amphibians” (“gefährden Vögel und Amphibien”) and specifically referred to protected amphibian, reptile, and bird populations (“europarechtlich geschützte Amphibien-, Reptilien- und Vogelbestände”), while authorising “targeted hunting” (“gezielte Bejagung”) [29].

Later Senate responses again used more qualified formulations. In November 2025, raccoons were described as “one cause” (“eine Ursache”) among multiple causes (“die Ursachen hierfür sind vielfältig”), while intensive hunting only “could” have a population-stabilising effect (“könnte sich … bestandsstabilisierend … auswirken”) [55]. In January 2026, whether hunting invasive species could contribute to protecting threatened animals (“ob eine Bejagung invasiver Arten zum Schutz gefährdeter Tiere beitragen kann”) remained subject to further assessment [47]. In February 2026, the Senate stated that “no specific data were collected” (“es wurden keine spezifischen Daten erhoben”) and that the effects of removing the closed season (allowing raccoons to be hunted year-round) would become apparent only “in the medium term” (“erst mittelfristig ablesbar”) [48].

The documents therefore show a shift from qualified, site-specific attribution toward stronger biodiversity-threat claims and regulatory intervention, followed by renewed qualifications concerning causal importance and management effectiveness.

## 4. Discussion

The findings show that raccoon management is shaped not only by ecological evidence and legal classification, but also by how harm, responsibility, and legitimate intervention are framed. The four frames identified in the corpus do more than describe different aspects of raccoon presence. They organise competing understandings of what requires protection: native biodiversity, urban order and property, the governability of raccoon populations, or the conditions of multispecies coexistence. This supports Entman’s account of framing as the selective connection of problem definitions, causal interpretations, moral evaluations, and remedies [21].

These frames also distribute responsibility differently. The invasive threat frame foregrounds predation and non-native status, locating the problem primarily in raccoon presence and its effects on protected species. Urban conflict and cohabiting neighbour framings make human practices more visible, including feeding, waste management, building design, and the historical introduction of raccoons. The manageable population frame shifts attention towards expert administration by rendering abundance and reproduction objects of technical intervention. Responsibility therefore moves between animals, residents, infrastructures, and public authorities according to how the problem is defined.

Terms such as “alien,” “invasive,” and “pest,” together with metaphors of invasion and warfare, can shape perceptions of ecological urgency and legitimate intervention [56,57,58]. Stanescu and Cummings similarly argue that invasion discourse heightens attention to “proper belonging and defense against the other” and treats invasivity as a politically non-neutral category [59] (p. viii). The frames likewise shape whose vulnerability becomes visible. Invasive threat discourse foregrounds amphibians, reptiles, birds, and other protected species, while the suffering of individual raccoons becomes less salient when animals are represented primarily as populations or predators. Animal-welfare and coexistence discourse reverses this emphasis by making capture, injury, killing, and surgical intervention more visible, but may give less attention to serious site-specific effects on vulnerable prey populations. The corpus therefore does not support a simple opposition between evidence-based conservation and ethically motivated animal protection. Rather, different framings select different harms for scientific, moral, and political attention.

This helps explain why the same legal classification, and sometimes the same ecological evidence, can support hunting, exclusion, fertility control, or coexistence. Terms such as “invasive”, “pest”, “management”, and “coexistence” connect empirical observations to broader narratives of risk, order, belonging, care, and control [56,57,58]. EU invasive-species law constrains the range of possible responses but does not determine which intervention is proportionate or preferable. Management choices therefore remain partly normative and political, even when expressed through scientific or administrative language.

The Berlin amphibian case illustrates how the policy significance assigned to ecological evidence can vary across institutional settings. Evidence of raccoon predation at particular sites is relevant to conservation, but it does not by itself establish equivalent population-level effects across Berlin [60,61]. The corpus shows movement between qualified descriptions of raccoons as a local or additional pressure and stronger formulations of biodiversity threat, while later Senate documents again acknowledged uncertainty about causal importance and the effects of expanded hunting. This does not imply that stronger threat claims were necessarily incorrect; rather, it demonstrates why spatial scale, interacting pressures, and evidential limits must remain explicit when ecological observations are translated into management. This is consistent with research emphasising the context dependence of invasive-species impacts and the role of conservation baselines and evaluative judgments in assessing harm [5,61,62].

Scientific authority was mobilised across competing positions. Hunting organisations, conservation actors, sterilisation advocates, and political parties all presented preferred interventions as scientifically grounded, although the evidential basis and degree of uncertainty were not always equally explicit. The 2025 Berlin ordinance also translated intervention into administrative necessity through the language of restoring “capacity to act” (“Handlungsfähigkeit”) where other measures offered “no acceptable solutions” (“keine vertretbaren Lösungsansätze”) [29]. This extends the findings of Mösch et al., who identified substantial differences among German actors in perceptions of raccoons and preferred management measures [15,19]. The present analysis shows that disagreement concerns not only empirical findings but also which evidence is considered relevant, what level of uncertainty is acceptable, and which harms justify intervention. Scientific evidence can establish predation, pathogen presence, population trends, or intervention outcomes, but it cannot independently determine whose harms should receive priority or when an intervention is proportionate. Management therefore remains partly normative even when expressed through scientific and administrative language.

Berlin and Kassel/Hesse also demonstrate that coexistence and control are not mutually exclusive governance models. Both cases combine prevention, modification of human behaviour, monitoring, case-based intervention, and population management. Berlin’s stronger emphasis on biodiversity protection after 2025 did not eliminate coexistence-oriented language, while Kassel’s sterilisation project offered an alternative to killing but retained population stabilisation and reduction as explicit objectives. The cases are therefore better understood as hybrid governance regimes in which different interventions coexist and compete for scientific, legal, and ethical legitimacy.

This complicates a simple distinction between lethal and non-lethal management. Capture, sterilisation, and release avoid deliberate killing but still involve capture, confinement, anaesthesia, surgery, recovery, and uncertain population-level effects. Hunting produces different welfare costs, including killing and possible wounding, while hunting-bag figures alone do not demonstrate long-term population reduction. The scale of intervention is nevertheless relevant: approximately 240,000 raccoons were reportedly killed in Germany in the 2023/24 hunting season, a figure the German Hunting Association used to question the practical feasibility of population-wide castration [45]. In Berlin’s settled areas, hunting is generally prohibited without justified special authorisation, which limits the use of firearms as an urban management method. From an animal-ethics perspective, however, the central question is not simply which technique controls raccoons most effectively or with the least suffering. It is whether raccoon abundance should be constituted as a problem of population control in the first place, whether the expected ecological benefits justify harms imposed on individual animals, and why invasive status is presumed to make those harms permissible. Effectiveness, feasibility, and proportionality become relevant only after the need for intervention has itself been justified; they cannot supply that justification merely because raccoons have been classified as invasive.

These competing approaches also operate at different ethical scales. Conservation arguments tend to foreground populations, species, and habitats, whereas animal-welfare arguments make the experiences of individual raccoons more visible. Each perspective foregrounds certain harms while making others less visible. Exclusive attention to individual animals may understate serious effects on vulnerable prey populations, while population-level reasoning can render the suffering of animals classified as invasive comparatively invisible [63,64,65]. The contrast shows that assessments of management depend on how ecological benefits, welfare costs, uncertainty, and feasible alternatives are weighted, rather than following automatically from invasive status or management method alone.

The Kassel sterilisation controversy further shows that management is shaped by multilevel governance as well as by scientific disagreement. The Hessian Hunting Association interpreted the release of sterilised raccoons as “expressly prohibited” (“ausdrücklich untersagt”) [26], whereas the Federal Environment Ministry stated that capture followed by sterilisation or castration and release could be considered (“in Betracht ziehen können”) as a management measure under Article 19, while leaving concrete decisions to the federal states [66]. The dispute therefore concerned not only biological effectiveness but also institutional competence and legal interpretation. Local authorities may identify and respond to specific conflicts, while the authority to approve particular interventions lies at another administrative level. This illustrates a broader tension in environmental governance between local responsiveness and fragmented regulatory competence.

The findings also reveal a tension between ecological establishment and discourse of belonging. The political significance of borders and belonging becomes especially explicit in the AfD inquiry, which contrasts the “alien and invasive species” (“gebietsfremde und invasive Art”) with “our native fauna” (“unsere heimische Tierwelt”) and invokes “homeland protection” (“Heimatschutz”) and “our long-established biodiversity” (“unsere angestammte Artenvielfalt”) [30]. Such language does not make wildlife classification equivalent to human nationalism or xenophobia, but it shows how biodiversity protection can be articulated through politically charged distinctions between borders, belonging, nativity, and intrusion. More broadly, Sinclair and Pringle argue that invasivity is not simply a neutral description but can organize questions of territory, belonging, and which animal lives become morally disposable [67].

Raccoons are treated in municipal guidance as permanent urban residents whose complete removal is unrealistic, yet they remain legally classified as invasive and therefore continuously available as objects of population control. The native/non-native distinction and invasive classification are thus scientifically and legally relevant, but they also organise relations of protection and exclusion [68,69]. The existing vocabulary within native/non-native discourse “relies on troubling definitions of closed ecosystems, neo-colonial borders, and anthropocentric taxonomies” [59] (p. vii). Long-term presence does not make raccoons native in a biogeographical sense, but neither does non-native origin determine the magnitude of local impact or the proportionality of a particular intervention. The movement from origin to intervention is therefore one of the paradigm’s normative operations: non-native status can function as a proxy for harm and as prior authorisation for control even where the magnitude and spatial extent of local effects remain contested [70,71].

The cohabiting neighbour frame broadens this responsibility by making human introduction, feeding, waste systems, buildings, and habitat transformation part of the causal account. Coexistence in this sense does not imply passive acceptance of all raccoon presence. It is itself a form of governance involving prevention, exclusion, tolerance, monitoring, and, where justified, targeted intervention [64,72]. The ethical significance of the cohabiting-neighbour frame is not that it supplies a preferred management technique but that it refuses to treat raccoon presence itself as sufficient evidence of a problem. It shifts causal and moral attention towards human introduction, conflict-producing infrastructures, and site-specific harms. It is not an ethically uncomplicated alternative, because prevention, exclusion, monitoring, and fertility control can also regulate and harm individual animals. Its critical value lies in reopening questions that the invasive-threat frame tends to settle in advance: what constitutes harm, who is responsible for it, and why raccoon lives become legitimate objects of control.

The study is limited by its focus on 39 publicly available documents and therefore captures articulated governance positions rather than policy implementation, institutional intention, public opinion, or ecological outcomes. Seven additional corpus documents were coded but are not discussed individually because they did not materially alter the identified frames or comparative patterns [73,74,75,76,77,78,79]. The purposive corpus is not statistically representative of German raccoon discourse, and differences in genre and institutional authority shape which positions become publicly visible. Future research should examine how discursive changes translate into practice, compare additional German cities, and combine document analysis with interviews, ethnographic observation, ecological monitoring, and comparative welfare assessment. In particular, the effectiveness and welfare consequences of hunting, fertility control, and preventive measures require longitudinal evaluation at appropriate spatial scales.

## 5. Conclusions

Raccoon governance in Berlin and Kassel/Hesse was shaped by a dominant invasive-species paradigm that organises ecological evidence, legal classification, and legitimate intervention. Ecological harms were central to the corpus, particularly evidence and claims concerning predation on vulnerable amphibians, reptiles, and birds at sensitive sites. However, the documents differed in their causal certainty and spatial scale: localised or potential effects sometimes became generalised threat narratives, while invasive status functioned as prior authorisation for management without determining or ethically justifying any single method. At the same time, human responsibility for raccoons’ introduction and for conflict-producing urban conditions, as well as the experiences of individual raccoons, received uneven attention. The fertility-control controversy further shows that non-lethal approaches do not necessarily stand outside the control-oriented paradigm or escape ethical scrutiny. The issue is therefore not merely how to manage an invasive species more humanely, but how the category of invasiveness constructs raccoon presence as a problem and narrows the range of legitimate responses towards control.

The central contribution of this analysis is to show that the invasive-species paradigm does not merely describe ecological relations but distributes moral visibility and defines the range of legitimate responses. By foregrounding populations, species, and habitats, it can render individual raccoons less visible as morally considerable beings and present their killing or removal as a technical necessity rather than an ethically contested choice. From an animal-ethics perspective, non-native origin and legal classification cannot by themselves diminish an animal’s moral status or determine in advance whether killing, removal, or reproductive control is justified. This does not deny documented predation on vulnerable animals or the possibility of population-level effects at sensitive sites; it shows that the magnitude and spatial extent of such effects must be demonstrated rather than presumed. Raccoon governance therefore emerges not as the straightforward application of ecological evidence to a predefined threat, but as a contested negotiation over evidence, responsibility, belonging, and whose lives are made protectable or disposable.

## Figures and Tables

**Table 1:** Analytical frames identified in the corpus and their implications.

Frame	Problem Definition	Causal Interpretation/Moral Evaluation	Treatment Recommendation
Invasive threat	Ecological danger to native species	Non-native status and predation justify protection of native biodiversity.	Containment, removal, targeted or intensified hunting.
Urban conflict/pest	Damage, nuisance, hygiene risk, or unwanted proximity	Urban conditions facilitate conflict; urban order and property should be protected.	Exclusion, food control, advice, case-based removal.
Manageable population	Excessive abundance or reproduction	Population growth is represented as administratively governable and requiring expert intervention.	Hunting, trapping, sterilisation, monitoring, adaptive intervention.
Cohabiting neighbour	Anthropogenic drivers of human–raccoon conflict	Humans contribute to conflict and bear responsibility for the species’ introduction and preventable encounters.	Prevention, education, building modification, tolerance, non-lethal response.

## Data Availability

The primary documents analysed in this study are publicly available from the institutional sources cited in the reference list. Document-specific coding sheets and working translations generated for the analysis are available from the author on reasonable request.
